# Implications of COVID‐19 in Airway and Swallowing Function

**DOI:** 10.1002/oto2.74

**Published:** 2023-09-08

**Authors:** Lauren Linquest, Kayla Ackerman, Karuna Dewan

**Affiliations:** ^1^ Department of Otolaryngology–Head and Neck Surgery LSU‐Health Sciences Center Shreveport Louisiana USA

**Keywords:** COVID‐19, discharge diet, dysphagia, prolonged intubation, SARS‐CoV‐2, swallowing function

## Abstract

**Objective:**

The acute treatment and complications of the novel COVID‐19 virus have been well studied, but the implications of this novel virus for swallowing function continue to be investigated. The goal of this study is to retrospectively assess airway and swallowing outcomes for those patients who required intensive care unit(ICU)‐level care for COVID‐19 infection.

**Study Design:**

Comparison of swallowing outcomes through diet change in COVID‐19 patients in the Ochsner‐Louisiana State University (LSU) Hospital ICU.

**Setting:**

Ochsner‐LSU Hospital (Shreveport, Louisiana).

**Methods:**

A retrospective chart review was performed from March 2020 to May 2022 to identify patients with a primary diagnosis of COVID‐19. Variables analyzed include age, gender, length of intubation, length of ventilation, airway interventions, use of extracorporeal membrane oxygenation, and diet prior to, during, and after hospitalization for COVID‐19 infection.

**Results:**

Two hundred and seven patients fit the inclusion criteria. There was a significant difference in discharge diet between those patients who were intubated and those who were not (*P* = .007). Thirty percent of patients were discharged on a different diet than their baseline with patients on a nonregular diet significantly more likely to discharge to a facility (*P* = .043). Negative vaccine status was associated with prolonged ICU stay, prolonged duration of intubation, and prolonged duration of ventilation.

**Conclusion:**

COVID‐19 continues to present novel challenges with new implications and outcomes being discovered in the third year of the pandemic. Further research is necessary to determine the most effective treatment approaches with respect to optimized speech and swallow outcomes.

Beginning in March 2020, the coronavirus pandemic swept the world, causing significant changes to our understanding of infectious disease.^1^ The upper respiratory infection caused by severe acute respiratory syndrome coronavirus 2 (SARS‐CoV‐2 or COVID‐19) is considered common and acute in nature; however, it can also result in life‐threatening multisystem infection.^2^ While 80% of patients have a mild infection with symptoms of fever, fatigue, dry cough, myalgia, dyspnea, and sputum production, 15% will develop severe disease marked by lung changes on imaging and 5% will become critically ill.^3^ Individuals most affected by severe disease are between 49 and 56 years of age with increased mortality in patients above the age of 80 and individuals with additional comorbidities.^3^ With the majority of patients being admitted to the intensive care unit (ICU) presenting with acute respiratory failure, the need to develop a protocol for airway management has become critical in coronavirus care.^4^

Severe coronavirus infection can have many manifestations that often significantly impact swallowing, voice, and motor speech. Some studies have found that as many as half of all COVID‐19 patients in the ICU will develop swallowing difficulties.^5^ This is worsened by the prolonged use of various invasive techniques that are required for airway management such as intubation and tracheostomy. Since the pandemic, the duration of respiratory support provided to coronavirus patients is significantly longer than viral pneumonia cases and the number of tracheostomies performed has risen as well.^6^ This was heightened in the early stages of the pandemic when guidelines had not yet been established, leading to extremely prolonged intubation times ranging from 21 to 35 days unlike the routine 7 to 10 days prepandemic.^7^ The utilization of tracheostomy procedures allows patients to be weaned off sedation, enables removal of the translaryngeal tube, and aids in avoiding resedation/intubation. However, the fear of aerosolization of viral particles during this procedure further exacerbated the duration of time patients remained intubated.^6^ These factors contribute to the range of postintubation sequelae experienced by coronavirus patients. Reports have listed as many as 41% of patients experiencing dysphagia following extubation.^4^ Previous studies before the coronavirus pandemic had also established this link, with moderate to severe dysphagia occurring in postintubated patients for 7 days or longer.^8^ This is in part due to laryngeal injury caused by intubation, leading to vocal cord paralysis, granuloma, and stenosis. Additionally, the neurological symptoms associated with SARS‐CoV‐2 infection can cause dysarthria, dysphonia, and dysphagia that is worsened by chronic disuse leading to muscle atrophy.^2^, ^9^ These procedures remain critical for coronavirus airway management; however, the effects on swallowing function and indications for intubation and early tracheostomy still remain unclear.

The goal of this study is to retrospectively assess the airway and swallowing outcomes for those patients infected with COVID‐19 who required ICU‐level care. Specifically, diet changes following hospitalization will be compared among hospitalized patients suffering from COVID‐19 infection.

## Materials and Methods

### Study Design and Population

**Figure 1:**
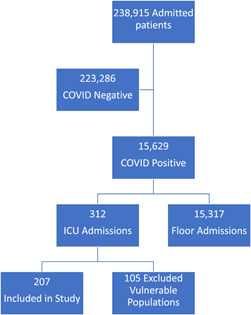
Flowchart demonstrating patient selection for inclusion in the study. ICU, intensive care unit.

All patients admitted to Ochsner‐Louisiana State University (LSU) hospital between March 2020 and May 2022 with a diagnosis of COVID‐19 were identified via retrospective chart review through the Epic medical record database (Epic Systems Corp. Verona, WI Version Feb 2023). Vulnerable populations were excluded. These are neonates, students, employees, pregnant patients, pediatric patients, non‐English speakers, cognitively impaired patients, and prisoners. Once identified, patients were further separated based on the need for upper airway intervention. Upper airway intervention was identified via a diagnosis of one of the following: acute respiratory failure, ventilator dependence, COVID‐19 pneumonia, dysphagia (all types), stridor, or shortness of breath. Patients excluded were individuals presenting to the hospital during the specified time frame without a diagnosis of COVID‐19. A flowchart demonstrating patient selection for the study is found in Figure 1. Formal approval and authorization were provided by the LSU Institutional Board Review approval prior to data collection. There was no risk to patients due as all data were collected retrospectively and confidentially.

### Data Collection

Through the electronic medical record, patient data was collected with a diagnosis of COVID‐19. Demographic information, medical co‐morbidities, vaccination status, and length of ICU stay were collected. Pre‐existing medical comorbidities prior to this current hospitalization were assessed through a retrospective chart review of the past medical history listed in this history of present illness performed on hospital admission. Information was further retrieved concerning airway management including airway interventions performed, duration of intubation, need for reintubation, tracheostomy status, use of noninvasive positive pressure ventilation, and any additional airway diagnosis given. The need for reintubation was defined as reintubation at any point during the hospitalization. To assess swallowing function and diet, an extensive review was performed on all speech‐language pathologist (SLP) notes provided for that patient during this current hospitalization. At this institution, the SLP team works extensively with the otolaryngology department for optimal patient care. In the SLP documentation during hospitalization, a preadmission diet was noted. Likewise, the discharge diet was collected from SLP documentation as well as from the discharge summary. Instrumental evaluation of swallowing was unable to be performed because of possible aerosolization of viral particles which was of particular concern during this stage of the pandemic. Furthermore, the American Speech‐Language‐Hearing Association (ASHA) deemed functional endoscopic evaluation of swallowing and barium swallow testing to also cause aerosolized particles.^10^ Because of this, these measures for swallowing function were unable to be analyzed. Through SLP documentation analysis, the functional oral intake score, a method of diet assessment, was calculated to aid in the statistical analysis of diet. Patient swallowing status and oral intake before, during, and after hospitalization was noted. Finally, any specific discharge needs for patients were also analyzed in this study.

### Statistical Analysis

Patient characteristics were analyzed using means, standard deviations, medians, frequencies, interquartile ranges, and percentages. A multivariable logistic regression model was created using a pre‐established covariate. *χ* ^2^ analysis was used to examine categorical variables. A 2‐tailed Student's *t* test was used to compare means. A Bonferroni correction for multiple comparisons was also performed.

## Results

### Subjects

**Figure 2:**
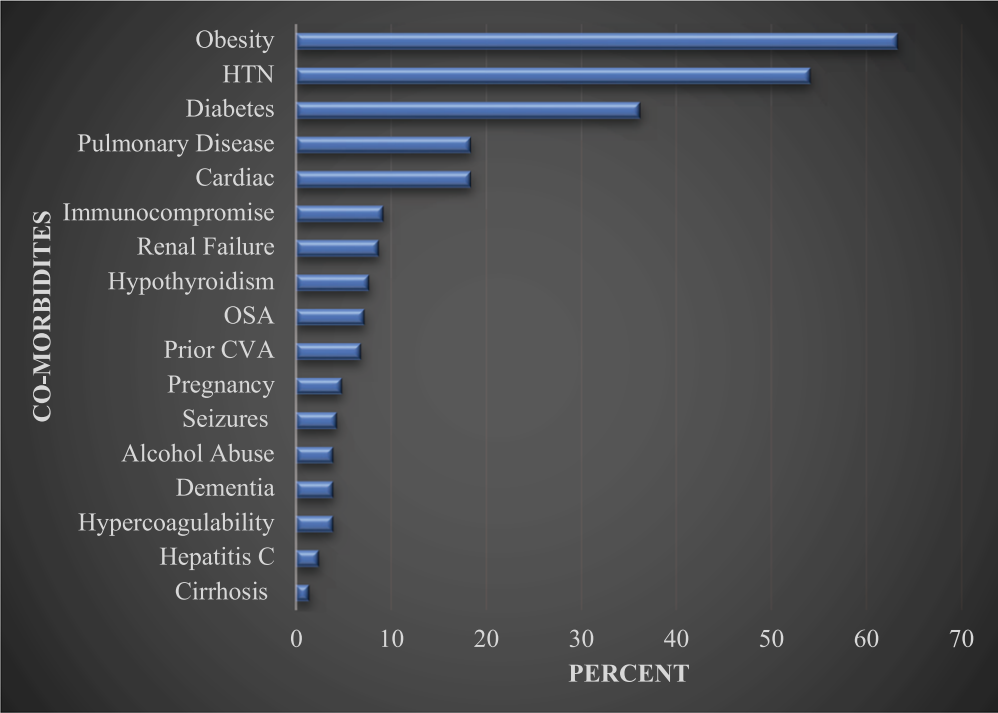
Distribution of co‐morbid conditions in the patient population. CVA, cerebral vascular accident; HTN, hypertension; OSA, obstructive sleep apnea.

A total of 207 patients were admitted from March 2020 to May 2022 time frame with a diagnosis of COVID‐19. Among these, 57% were male and 8.7% were vaccinated. The average age was 55.6 ± 15.9 years and the average length of stay was 14.7 ± 16.9 days. Of these patients, 63.4% required intubation at some point during their ICU stay with the average duration of intubation being 7.7 ± 9.7 days (Table 1). Furthermore, 29 of the 207 total patients (14%) required tracheostomy (Table 1). At discharge, 3.8% still needed ventilatory support. The most common pre‐existing medical comorbidities diagnosed prior to this hospitalization included hypertension (54%), diabetes mellitus (36.2%), and obesity (29.9%). A complete list of pre‐existing medical comorbidities is found in Figure 2. A total of 92 of the 207 patients died and 115 were alive at discharge.

**Table 1:** Airway Management in COVID‐19 Patients Requiring ICU Care

Airway management	
Duration of intubation	7.7 ± 9.7 d
Duration of assisted ventilation	9.7 ± 14.4 d
Intubation rate	63.4%
Extubation failure rate	13.6%
Requiring ventilatory support on discharge	3.8%
Tracheostomy rate	14%
Decannulation	15%

Abbreviation: ICU, intensive care unit.

### Airway Management

**Figure 3:**
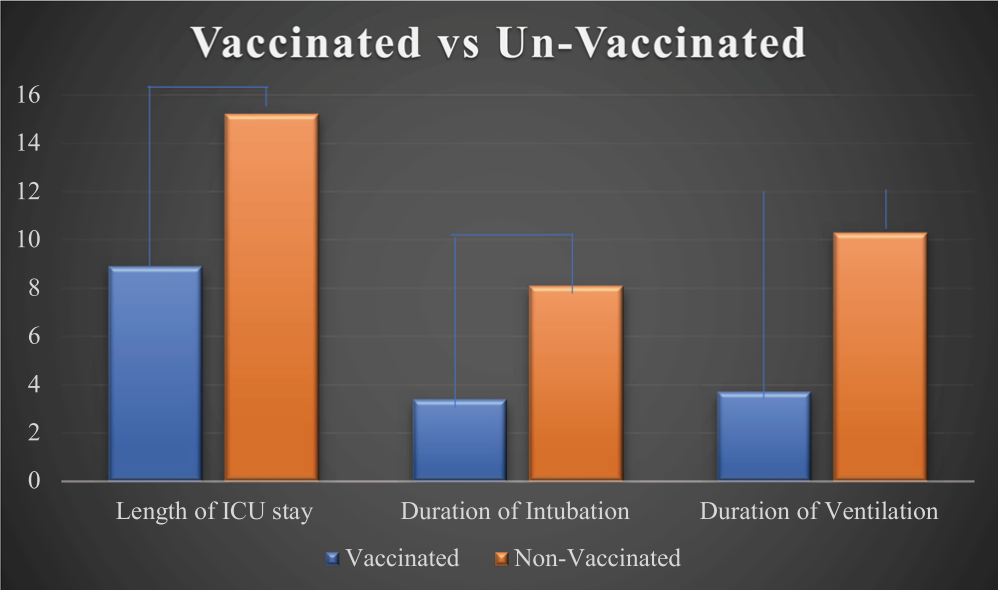
Comparison of the duration of the length of stay, duration of intubation, and duration of ventilatory support in patients with and without COVID‐19 vaccination. ICU, intensive care unit.

**Figure 4:**
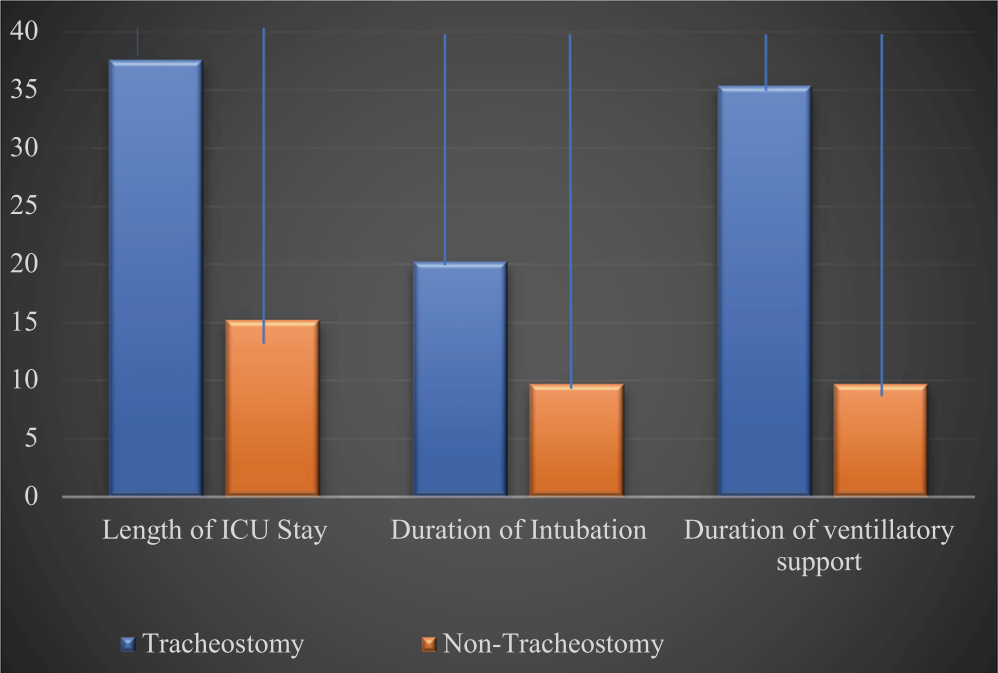
Comparison of the duration of the length of stay, duration of intubation, and duration of ventilatory support in patients with and without a tracheostomy. ICU, intensive care unit.

Advanced age was significantly related to intubation requirement with the mean age of those intubated being 59.14 ± 17 years (*P* = .02). Additionally, prolonged ICU stay was also associated with intubation with most intubated patients staying an average of 20.9 ± 19 days (*P* < .01). Negative vaccine status was associated with prolonged ICU stay, prolonged duration of intubation, and prolonged duration of ventilation but was not significantly related to death (Figure 3). Furthermore, patients with a history of cardiac disease (*P* = .014), diabetes mellitus (*P* = .037), hypercoagulable state (*P* = .019), immunocompromise (*P* = .021), seizures (*P* = .019), and dementia (*P* = .006) prior to this hospitalization were significantly more likely to be intubated. Of the intubated patients, 13.5% failed their first attempt at extubation and had to be reintubated (Table 1). Patients requiring tracheostomy experienced a significantly longer ICU stay than the intubated patients who did not undergo tracheostomy tube placement. Patients who underwent tracheostomy placement also had a significantly longer duration of intubation than those who didn't undergo tracheostomy tube placement. And finally, patients who required tracheostomy tube placement had a significantly longer duration of mechanical ventilation than those without a tracheostomy (*P* < .01 for all) (Figure 4). When analyzing surviving patients, among the 29 patients who received a tracheostomy, 5 (17%) were decannulated before discharge (Table 1). Lastly, the patients who died were significantly more likely to have been intubated for longer (*P* < .01) and ventilated for longer (*P* < .01). Patients who were intubated were also significantly more likely to die (*P* < .01). However, there was no significant difference found in age, duration of ICU stay, or body mass indexbetween those who died and those who did not.

### Diet

When comparing the diet change of the 115 patients that survived, a significant difference was found in the discharge diet between patients who were intubated and those who were not (*P* = .007). Furthermore, patients that were intubated and those that had a tracheostomy were significantly less likely to be discharged on a regular diet. Among intubated patients, those that were NPO had a significantly prolonged duration of intubation (13.2 ± 13 days vs 3.7 ± 7.4 days) and significantly longer ventilation (20.3 ± 19.7 days vs 4.11 ± 8.43 days) (*P* < .01, *P* < .01). Patients that died were significantly more likely to have been on a diet other than normal during their hospital stay. Of the surviving patients, a total of 30% of patients were discharged on a diet that differed from their baseline diet, as seen in Table 2, with patients on a nonregular diet significantly more likely to discharge to a facility (*P* = .043).

**Table 2:** Diet Management in the Cohort of COVID‐19 ICU Patients

Diet metrics overall	
Diet on discharge	
Same as admission	69.6%
Different from admission	30.4%
PO diet on discharge	
Yes	80.9%
No	19.1%

Abbreviation: ICU, intensive care unit.

### Discharge posthospitalization

**Figure 5:**
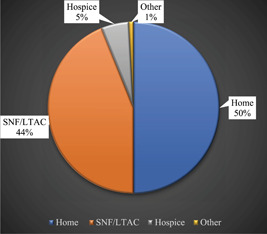
Distribution of discharge location following hospitalization in the studied patient population. LTAC, long term acute care facility; SNF, skilled nursing facility.

Discharge to a facility was associated with prolonged duration of ICU stay, prolonged duration of intubation, and prolonged duration of ventilation (*P* < .01 for all) (Table 3). Additionally, patients who were evaluated by SLP were more likely to discharge to a facility (*P* = .043). No significant difference was found in the pre‐COVID‐19 diet between patients who went to a facility compared to being discharged home. Patients who were ultimately discharged to a facility were significantly older as well. The distribution of discharge locations is seen in Figure 5.

**Table 3:** Discharge Disposition in COVID‐19 ICU Patients

	Facility	Home
Duration of ICU stay	21.1 d	7.6 d
Duration of intubation	9.3 d	1.9 d
Duration of ventilation	12.5 d	2.05 d
Tracheostomy placement	28.1%	1.7%
Average age	58.2 y	49.9 y

Abbreviation: ICU, intensive care unit.

## Discussion

The coronavirus pandemic continues to shift our understanding of airway management in the critical care setting and the effects of invasive management. Among the many sequelae of prolonged intubation, this study found that diet is directly impacted by this form of care. Through this retrospective chart review, a significant association between prolonged intubation and the need for tracheostomy, modified diet at discharge, and discharge to an outside facility was elucidated.

It has been well known that a longer duration of intubation and delayed time to tracheostomy increases rates of laryngeal complications.^9^ Since the COVID‐19 pandemic, these rates have amplified and brought on significant concern in the otolaryngologic community about posthospitalization outcomes.^11^ Among SARS‐CoV‐2 patients admitted to the ICU in our study, 63.4% needed to be intubated with an average duration of intubation of 7.7 ± 9.7 days. Hur et al found similar results in their population when assessing for factors that contribute to prolonged intubation in COVID‐19 patients. In this article, only 27.5% of patients were intubated for less than 7 days with 90% having a prolonged hospital stay >10 days.^12^ These findings of prolonged intubation times are why the European Laryngological Society issued an article to notify physicians of the high likelihood of increased sequelae being seen in COVID‐19 patients. In this report, concern over enlarged and overinflated cuffs to decrease virus spread was discussed. According to the committee, overinflation of the cuff can result in ischemic damage to the airway mucosa. Additionally, the endotracheal tube lays on the posterior aspect of the larynx, resulting in chronic pressure of the cricoarytenoid joints, posterior commissure, and cricoid plate. Combined, this can cause a range of adverse effects including granulomas, webs, laryngotracheal stenosis, tracheomalacia, tracheal necrosis, and fistulae development.^11^ A longitudinal cohort study performed by Lindh et al evaluated intubated COVID‐19 patients in the hospital for dysphagia using a bedside swallowing evaluation following extubation. They found that 71% of patients had dysphagia. These patients presented with symptoms of pharyngeal muscle weakness, cough, and bolus retention. While 47% of patients did recover oral intake at discharge and nearly all had complete resolution by their clinic follow‐up, this impairment remains an important concern in the hospital setting.^9^ To reduce the risk of in‐hospital dysphagia and possible long‐term side effects, early tracheostomy is indicated.^11^ Out of our cohort, 14% required a tracheostomy. The intubated individuals who did not get a tracheostomy were found to have a significantly longer ICU stay, duration of intubation, and duration of ventilation. This stresses the importance of performing an early tracheostomy to decrease the prolonged need for ventilatory support in hopes to prevent airway sequelae.

As described, the prevalence of dysphagia is a significant concern among COVID‐19 patients who underwent intubation. Various parameters can indicate a patient's swallowing function, including discharge diet. This study found that 30.4% of patients had to be discharged on a diet different than the diet at admission. Furthermore, no significant difference was discovered in the discharge diet between those patients who were intubated compared to those that were not. In the study performed by Lindh et al, their patients experiencing dysphagia were more likely to be older, have invasive ventilation or tracheostomy longer, and have prolonged ICU and hospital stays.^9^ Another study by Zannidi et al assessing weight loss in hospitalized patients found that increased length of stay was an independent risk factor for >10% weight loss in patients.^13^ With an average duration of 14.7 ± 16.9 days in our cohort, the ability for oral intake is a significant concern in preventing malnutrition. In‐hospital malnutrition can further exacerbate the disease burden and increase mortality.^14^ This is demonstrated by our finding that NPO status in intubated individuals resulted in a significantly prolonged duration of intubation and ventilation. Zannidi et al study patients with significant weight loss also had markers for worse disease severity including higher rates of oxygen therapy, ICU admission, mechanical ventilation, and dysphagia, in addition to others.^13^ Furthermore, a study in France found that even 30 days after discharge patients remained malnourished, which could be predicted by the use of mechanical ventilation while hospitalized.^15^ Lindh et al found a moderate association between the presence of dysphagia and patient discharge to a home or rehab clinic.^9^ Our study found a significant association between age, prolonged ICU stay, duration of intubation, and duration of ventilation with the need to discharge to a facility. Combined, an increased level of suspicion should be taken in our patients who are intubated and in the ICU as these individuals may have a high risk of dysphagia and malnutrition and require prolonged supportive care. After discharge, COVID patients continue to have prolonged mental and physical changes. A recent article by Martillo et al reported rates of postintensive care syndrome, the presence of any impairment affecting the physical, psychiatric, or cognitive domains because of critical illness, in COVID‐19 patients. They found that 91% of COVID‐19 ICU survivors met the criteria for PICS symptoms with 87% exhibiting a physical impairment in addition to mental health sequelae like depression, posttraumatic stress disorder, and insomnia.^16^ This can directly affect the quality of life in patients with COVID‐19, especially those who were admitted to the ICU.

A secondary finding in this study was the role vaccination played in‐hospital outcomes. In December 2020, the first available vaccine for SARS‐CoV‐2 became available to the public. This was a breakthrough in the science community. After the availability of the vaccine, vaccinated and unvaccinated individuals and their hospital stays were compared. This study found that unvaccinated individuals had a significantly longer ICU stay, longer duration of intubation, and longer duration of ventilation. However, there was no association between vaccination status with increased risk of death found. An extensive case‐control study performed by Tenforde et al included 4513 hospitalized adults across 18 US states to understand the correlation between vaccination status and disease severity. They found that unvaccinated patients accounted for 93.9% of cases that progressed to death or invasive mechanical ventilation. Additionally, patients that were vaccinated yet still developed COVID‐19 (termed a breakthrough case) had a significantly lower level of disease severity as compared to unvaccinated individuals.^17^ This information, combined with our results, suggests that unvaccinated individuals have a higher risk of severe COVID‐19 infection and that vaccination may be able to decrease this risk. Mortality remains to be more extensively evaluated to determine its correlation with vaccination status. Other studies in Greece^18^ and Norway^19^ have had varied results. Grapsa et al found significantly lower mortality but no significant difference in length of ICU or hospital stay, while Whittaker et al did not find an association with in‐hospital death but did find a significantly shorter length of stay and lower risk of ICU admission in patients 16 to 79 years old.^18^, ^19^ These discrepancies may be due to different patient populations in these countries compared to the United States and the types of vaccines used.

This study has several limitations. The study did not use a standardized parameter to evaluate swallowing function pre‐ and postintubation. This limits the ability to draw direct conclusions about why patients had a different discharge diet. Patients' swallowing function and diet following discharge were also not evaluated. This information would be useful in understanding the long‐lasting effects of prolonged intubation.

In conclusion, COVID‐19 is a significant public health concern, causing serious illness in a subset of patients that can contribute to changes in upper airway function, resulting in sequelae like dysphagia and alterations in diet.

## Author Contributions

**Lauren Linquest**, draft manuscript preparation, and data analysis; **Kayla Ackerman**, study conception and design, data collection, analysis, and interpretation of results; **Karuna Dewan**, study conception and design, data collection, analysis, and interpretation of results, and draft manuscript preparation.

## Disclosures

### Competing interests

None.

### Funding source

None.
